# Transforming surgical care and safety: dissecting the impact of checklists in the global south

**DOI:** 10.3389/fsurg.2025.1664277

**Published:** 2025-11-17

**Authors:** Chaitanya Reddy, Lovenish Bains, Padmavathy Krishna Kumar, Siddhesh Zadey

**Affiliations:** 1 Association for Socially Applicable Research (ASAR), Pune, India; 2 Department of Surgery, Maulana Azad Medical College, New Delhi, India; 3 Columbia University Mailman School of Public Health, New York, NY, United States; 4 Dr. D. Y. Patil Dental College and Hospital, Dr. D. Y. Patil Vidyapeeth, Pune, India

**Keywords:** surgical safety checklist, low- and middle-income countries, global south, implementation, morbidity and mortality, surgical outcomes, barriers, facilitators

## Abstract

Surgical errors and preventable harm remain major public health concerns, especially in the low- and middle-income countries (LMICs). The World Health Organization's Surgical Safety Checklist (SSC) was developed as a low-cost, high-impact tool to improve surgical outcomes and enhance patient safety. This review examines how the SSC functions both as a safeguard against preventable errors and as a driving force for reducing morbidity and mortality in surgical care. Drawing on evidence from LMICs, we examine the checklist's impact on reducing surgical errors, associated complications, morbidity, and mortality, while also fostering better team communication and accountability in operating rooms. Despite SSC's proven benefits, its implementation in LMICs remains inconsistent due to barriers such as hierarchical team dynamics, limited training, infrastructure gaps, and lack of leadership support. The article highlights approach such as including structured training programs, hands-on demonstrations, workshops and the use of digital tools and platforms for better SSC implementation. It also emphasizes the role of local champions, leadership endorsement, local adaptations and regular audits with feedback to sustain adherence and foster a culture of surgical safety. Strengthening these efforts can transform the SSC from a procedural formality into a powerful tool for surgical safety, providing a practical pathway to enhance patient safety and quality in global surgical care.

## Introduction

‘First, do no harm’ is a universal core principle of clinical practice. Yet, the harsh reality is that globally, up to 83% of harm in clinical contexts such as adverse events and associated mortality are preventable (1). Unsafe healthcare practices rank among the top ten global causes of death, with the Global South bearing the brunt of the burden. According to the World Health Organization (WHO) Patient Safety data, surgical errors account for 10% of all preventable harm while diagnostic errors contribute 5%–6% (2). The Global South, with its large population, bears a disproportionately high surgical case burden. Each year, an estimated 5.7–8.4 million deaths are attributed to poor and unsafe surgical care (3). In low- and middle-income countries (LMICs), unsafe surgical procedures occur nearly three times more often than in high-income countries (HICs), with approximately 25% of all surgical care considered unsafe (4).

SSC is a simple tool that can improve surgical safety and quality. This narrative review investigates the dual role of the WHO Surgical Safety Checklist (SSC) as both a 'safety net’ and a ‘catalyst for reducing surgical morbidity and mortality’ in LMICs. In places where medical resources and staff may be limited, SSC helps ensure that every surgery follows the same safety steps. This reduces errors, improves teamwork and saves lives. Regular use of the SSC can improve patient outcomes and strengthen trust in the healthcare system by gradually transforming surgical care (3). The impact is particularly pronounced in general surgical procedures including appendicectomy, hernia repair, cholecystectomy, and laparotomy, reflecting the substantial baseline procedural volume in LMICs (4). It also shows a significant improvement in checklist use and adherence over 15 years. Globally, the implementation of the SSC has yielded mixed outcomes; however, substantial evidence supports its role in reducing surgical morbidity and mortality (5). This article examines how the SSC is helping to bridge quality-of-care gaps in LMICs and evaluates its potential and effectiveness in reducing complications through safer surgical practices. We dissect barriers such as entrenched hierarchies and workforce shortages, while highlighting emerging approaches. It outlines a roadmap for strengthening the impact of the SSC by aligning global standards with local realities. In doing so, the review calls for a paradigm shift recasting the SSC from a bureaucratic formality into a catalyst for systemic change capable of transforming surgical care for the world's most vulnerable populations.

## Surgical safety in the global south

While billions lack access to surgery when they need it, even those with access often face suboptimal outcomes due to quality and safety gaps (6, 7). Globally, of the 234 million surgeries performed annually, approximately seven million results in adverse events and one million patients succumb to complications during or shortly after surgery (5). The Joint Commission International's (JCI) 2023 Review highlights that wrong-site surgeries and retained foreign objects each accounted for 8% of 1,411 sentinel events, ranking second and third. Compared to 2022, wrong-site surgeries rose by 26% and retained object incidents by 11%, highlighting the urgent need to strengthen surgical protocols and team communication (8). Even after more than two decades since the launch of JCI, which mainly provides data on HICs, errors still occur. Contributing factors often involve poor adherence to safety protocols, ineffective communication within surgical teams, workforce fatigue, and persistent shortages of staff and resources (9–11). These challenges are further amplified in districts, peripheral hospitals or resource-limited settings where limited specialist availability and higher patient workloads significantly elevate the risk of adverse events (12).

Recent studies show surgical outcomes in LMICs are far worse than in HICs, with 30-day mortality after gastric or colorectal surgery three to four times higher and pediatric mortality ten times higher (13, 14). Perioperative complications, including surgical site infections are also more common and LMIC patients face two to three times greater odds of death or major complications (15). Postoperative morbidity and mortality remain disproportionately high across the Global South, reflecting systemic deficiencies in surgical safety and infrastructure (16). A systematic review on abdominal surgical emergencies in Sub-Saharan Africa reported an overall postoperative morbidity of 24.2%, including surgical site infections (SSIs) at 14.4% and a 30-day mortality of 7.3% (9). The GlobalSurg collaborative reported SSI rates of 23.2% in low-HDI (Human Development Index) countries vs. 14.0%in middle-HDI and 9.4% in high-HDI settings (10). Although overall morbidity rates after cancer surgery appear similar across income groups, 30-day mortality remains markedly higher in LMICs, largely driven by major complications, emergency presentations, and inconsistent implementation of safety practices, one of them being WHO checklist (11, 13).

## Surgical safety checklist

**Figure 1:**
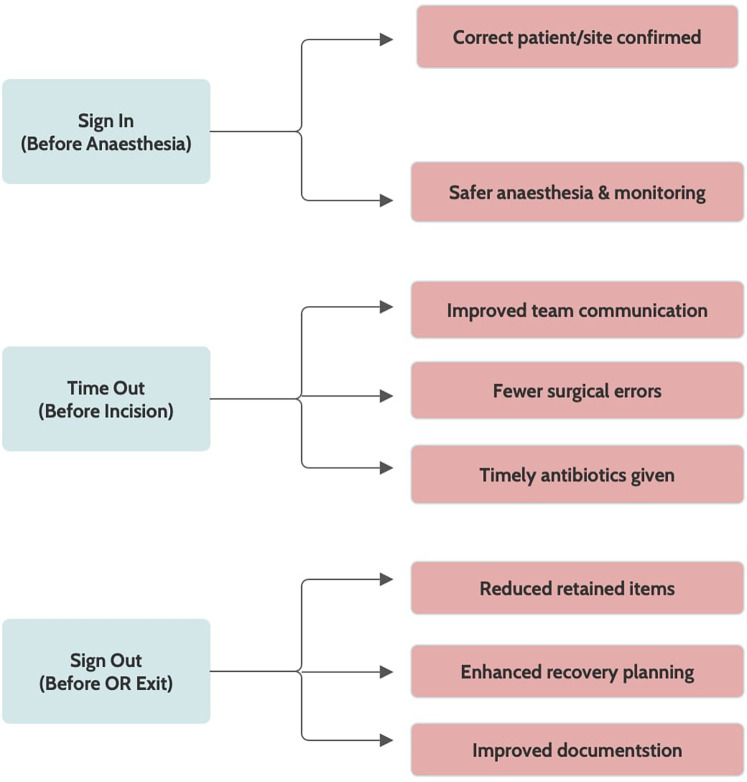
WHO surgical safety checklist (SSC) components and its impact.

The WHO developed the SSC to reduce errors and enhance patient safety globally. It is a 19-item tool designed to strengthen teamwork and ensure critical safety steps across all phases of surgery, thereby reducing errors and adverse events (12). The SSC is structured into three checkpoints. The first, sign in (before induction of anesthesia), verifies elements such as surgical site marking, anesthesia safety, equipment functionality, instrument counts, and specimen labelling. The second, time out (prior to skin incision), emphasizes team confirmation and the administration of prophylactic antibiotics within 60 min to lower the risk of surgical site infections. The final phase, sign out (before the patient leaves the operating room), involves reviewing the procedure performed, reconciling instrument and sponge counts, and highlighting key considerations for postoperative recovery (17). Notably, equipment malfunctions remain a significant contributor to intraoperative errors, underscoring the importance of thorough equipment checks during implementation (18) (Figure 1).

## Efficacy of SSC

A decade ago, surgical safety in LMICs faced high complications and mortality rates which were avoidable. Major surgeries had death rates of 5%–10%. Despite low operative volumes, high case-fatality persisted. In 2008, implementation of SSC across eight hospitals reduced major postoperative complications from 11% to 7% and perioperative mortality from 1.5% to 0.8% (6). By 2021, meta-analyses reported a 44% reduction in overall complications and a 23% decrease in mortality, indicating persistent effectiveness at scale (19, 20). Initial studies also showed low compliance rates with critical items often skipped due to time pressure, unclear roles or staff resistance (21, 22). In contrast, in 2024 studies reported near-complete adherence with all essential components of the checklist consistently completed, including patient identity verification, surgical site confirmation, procedure checks and anesthesia safety (23).

The SSC has been linked to 47% reduction in surgical mortality (24) and a 36% decrease in postoperative complications (2) and its adoption has shown promise in bridging quality and safety gaps despite systemic challenges in LMICs. Staff use of the WHO SSC was studied across 135 surgeries in Vietnam, with compliance rates ranging from 77% to 93% which led to reduction in errors (25). SSC implementation also reduced surgical complications and improved team communication in Thailand, underscoring its critical role in enhancing patient safety and the need for wider adoption (26). A nationwide survey across 172 health facilities in Ethiopia found that surgeries adhering to the WHO SSC had a statistically significant reduction in perioperative mortality and anesthesia-related adverse events. However, only 60.8% of the checklists were filled completely and correctly, indicating the need for improved compliance to maximize patient safety (27, 28). A quality improvement project in Uganda focusing on the WHO SSC and surgical counts led to significant enhancements in compliance. Checklist adherence increased from a median of 29.5% to 85% and surgical count compliance rose from 25.5% to 83%. These improvements were associated with a reduction in surgical complications demonstrating the impact of consistent use (29). Similarly, the SSC was used in 87.25% of 102 surgeries in Sri Lankan hospitals citing good adherence (30).

A modified WHO SSC in India significantly reduced postoperative complications and mortality where wound-related complications, abdominal complications, and bleeding complications were notably lower in the checklist group (31). Pakistan's SSC usage rose from 20.4% to 89.9% over four years, resulting in a 56.9% reduction in SSIs. (32) Overall checklist completeness in Rwanda improved from 78.3% at baseline to 86.3%, 92.0%, and 94.7% following the first, second, and third interventions, respectively. This stepwise increase in adherence was associated with fewer errors and complications suggesting that consistent use of SSC directly enhances surgical safety (33). In a team-based approach across 40 health facilities in two regions of Tanzania, self-reported checklist use rose from 0% at baseline to 98% by the end of the year. Additionally, the completeness of the checklists improved from 82.1% to 92.8%, particularly in health centers compared to hospitals (34). These findings highlight that consistent SSC use across LMICs improved surgical outcomes, strengthened teamwork and fostered a culture of patient safety.

Despite SSC's proven benefits, implementation challenges persist in LMICs due to non-standardized protocols, inadequate infection control practices, poor infrastructure, inadequate surgical staff and unequal access to surgical care (35). In this context, introducing SSC into resource-constrained settings can play a crucial role in reducing the impact of healthcare workforce shortages by standardizing procedures and improving team communication. While checklists do not replace trained personnel, they enhance the efficiency and reliability of existing teams.

## SSC implementation landscape

Use of the WHO SSC remains uneven in many resource-constrained settings despite healthcare professionals being aware of the benefits of using the checklist (36). Inadequate implementation of the SSC in Egypt was observed in 100% of high-volume cases and 69.4% of cases involving patients with chronic diseases due to their heavy burden (37). A total of 320 surgical procedures was analyzed in Nigeria, with 134 undergoing direct observation for checklist implementation, revealing a utilization rate of 96.9% (38). Participants in a descriptive qualitative interview in Indonesia acknowledged the positive impact of SSC on patient safety; however, challenges such as compliance issues, teamwork dynamics and unsafe behaviour persisted (39). Observational study in the Indian government setups highlighted that SSC was used by 83.5% of surgeons, 16.1% of anesthetists, and only 0.4% of nurses whereas in National Accreditation Board for Hospitals and Healthcare Providers (NABH) accredited hospitals it is being followed mostly by nurses (40). The variation in checklist usage across cadres may be due to hierarchical dynamics, role clarity, and training gaps in the OR. Surgeons, often leading the surgical team, are more likely to initiate and take responsibility for checklist use, especially since surgical outcomes are closely associated with their performance (41). Additionally, inconsistent training, poor interprofessional communication, and lack of institutional enforcement of checklist protocols can further contribute to uneven adoption among the team (36).

Similar problems in routine adoption of the SSC exist across several other LMICs as well. For example, a survey found that only 25% of anesthesia teams in East African referral hospitals regularly used the SSC. Two major hospitals, one in Uganda and one in Burundi, did not use the SSC because it was not available (42). However, evidence shows that training can make a big difference. In Somali hospitals, an education program led to an increased use of SSC from 37% to 99% of surgeries. However, complication and mortality rates were not investigated due to resource-limited settings (43). These variations highlight how leadership, training, and resource availability influence checklist use. In addition, a recent review confirmed that checklist adoption improves outcomes and enhances teamwork when it is fully implemented after making locally relevant modifications (44). A national survey in Senegal found that only one-third of hospitals used the WHO SSC in pediatric surgery. The main barriers were lack of training and access to the checklist, suggesting that targeted interventions could improve compliance and surgical safety (45).

A Malawi initiative uses the SSC with the Clean Cut framework to improve OR safety and train staff, strengthening six key perioperative practices such as skin preparation, antibiotic prophylaxis, sterile field maintenance, instrument sterility, gauze counting and SSC use, leading to a 35% reduction in SSIs across multiple countries. For example, in Blantyre the framework has reduced wound infections by over 30% (46). These efforts help make surgeries safer and strengthen health systems for the future. Similarly, the National Patient Safety Implementation Framework (NPSIF) for the years 2018–2025 was introduced by the Ministry of Health and Family Welfare in India in order to identify the necessity to reduce errors and prioritize patient safety. Despite its comprehensive vision, the framework faces several persistent challenges. These include limited awareness about patient safety practices, underreporting of adverse events due to a culture of blame, insufficient training, and lack of infrastructure especially in resource-limited settings (47).

## Implementation barriers

Policymakers may assume the checklist is used correctly (work-as-imagined), but frontline staff, especially in resource-limited settings, often face time pressure and staff shortages, leading to inconsistent use (work-as-done) (48). Most teams in Sri Lanka attach the checklist but often do not fully complete it: only ∼34% of checklists were fully filled out, and senior consultants participated in just ∼7% of cases (30). Entrenched hierarchies in Ethiopia have been cited as key obstacles where for example, studies note ‘hierarchical surgical team structure’ and ‘lack of ownership from seniors’ as factors impeding SSC use (49). Similarly, in Pakistani ORs, high surgical volume and time pressure lead staff to rush or skip checklist steps; interviews described surgeons ‘hastening the remaining procedure, not giving nurses enough time to do everything appropriately.’ (32, 50) A qualitative systematic review between 2022 and 23 (51) identified many persistent barriers: hierarchy (senior surgeon resistance), staff training gaps, low engagement, perception of redundancy (“we already do safety”), workflow interferences, lack of feedback/audit. Additionally, lack of printed checklists, perceptions of extra work and staff believing it is not their responsibility to perform checks hindered consistent adherence and effective SSC use in the OR (52). Interestingly, a critical gap was also noted in Rwanda between pre-operative, intra-operative and post-operative SSC implementation despite adequate awareness about its effectiveness. Implementation of and adherence to the checklist was influenced by job title and clinical experience which targeted specialized training (33). Similarly, in Zambia, the first barrier was rooted in team hierarchies and inadequate training which hindered SSC's intended use. The second was related to resource and logistical constraints that affected SSC introduction. Third was the high volume of cases. These structural barriers limit consistent application despite adequate awareness (53).

Language and cultural mismatches can further impede uptake—for instance, early SSC pilots in India translated the checklist into local languages and adapted it culturally to improve acceptance (24). However, due to the lack of a formal training period, these approaches lead to inconsistent knowledge levels with some viewing the checklist as a bureaucratic formality rather than a vital safety tool (36). Whereas, in a teaching hospital of India, only 30% of healthcare workers had received any formal SSC training (40). Inadequate training leads to lack of team coordination and communication which are key elements for successful WHO SSC execution. Without proper orientation, team members may view the checklist as a formality despite local adaptation to enhance patient safety (40). The paucity of structured training programs is evident in studies from LMICs. In North India operating surgeons, interns, operation theater staff, perioperative nurses, anesthetists and nurse anesthetists primarily learned about the SSC through the Internet (40) while in South India 70% of surveyed operating surgeons, nurses, anesthetists working in district hospital at Karnataka reported only partial knowledge of checklist procedures despite undergoing training (54). These gaps show the urgent need to include the WHO SSC in standard training programs. Tailored training courses aimed at improving patient safety can help remodify the training gaps as they allow all team members to practice SSC use in real-time (55). Peruvian hospitals noted to have a low perception of patient safety culture with key barriers including a punitive response to errors and insufficient staffing levels. This highlights the lack of a strong safety culture among healthcare professionals in the region, creating a major barrier to better surgical outcomes (56).

## Research gaps and recommendations

Despite growing recognition of the broader economic and social consequences of patient harm such as increased healthcare costs, disability and reduced productivity, LMICs research on surgical safety and the effectiveness of the WHO SSC remains limited (57). For example, the overall compliance of SSC implementation across LMIC was found to be suboptimal. Compliance rates were particularly low with many items either omitted or inconsistently used. This indicates that awareness alone did not guarantee effective use (58, 59). While quantitative data demonstrate reductions in complications when the checklist is used, there is a lack of in-depth qualitative research exploring the underlying reasons for inconsistent adherence. Understanding contextual, cultural and organizational barriers in LMICs through qualitative studies is essential to design targeted interventions and enhance compliance (60, 61).

Multi-modal strategies are crucial for tailoring interventions to LMICs unique socio-cultural and resource constraints as well. Regular refresher courses and performance audits can help sustain adherence and identify areas for improvement (62). Refresher courses focus on continued learning, ensuring that all members of the surgical team remain updated on checklist protocols, understand their roles and are reminded of the checklist's importance in improving patient safety (63). These sessions also offer opportunities to address misconceptions, strengthen communication skills, and reinforce a culture of safety. On the other hand, performance audits involve systematically reviewing how the checklist is being used in practice for identifying gaps in compliance, variations in implementation across departments, and opportunities for improvement. Audits provide objective data that can guide targeted interventions. Implementation science can further provide valuable insights into how training and monitoring efforts can be optimized in diverse LMIC healthcare settings. Future research should also focus on implementation costs in maintaining WHO SSC use. Understanding the cost of training, staffing time, and monitoring is crucial, especially in resource-limited settings. Without clear data on expenses, it is difficult to plan for large-scale or long-term use. Efforts are underway to support safer surgical practices and build stronger systems for the long term (44). Lifebox reports that it has trained over 12,000 providers worldwide on safer surgery and SSC use (46).

Lastly, Implementation science has emerged as a critical discipline to bridge the gap between evidence-based interventions (EBIs) and their consistent application in real-world clinical settings; however, the limited use of its methodology in LMICs represents a missed opportunity to effectively connect research, policy, and practice (64, 65). This methodology helps move beyond asking *does the SSC work?* to *how to make SSC use real, sustained and effective in LMIC contexts* (66). A shift in focus from individual clinical outcomes to organizational and systemic processes will allow for a deeper understanding of barriers and facilitators within healthcare environments.

## Strategies to improve SSC implementation

Transforming surgical safety culture in LMICs requires a multi-pronged approach. An example from rural Ethiopia shows that context-specific quality improvement projects have effectively increased SSC use achieving full (100%) adherence (67). Healthcare institutions must therefore prioritize regular and structured training programs for all OR team members. These programs should not only focus on technical aspects but also address interpersonal dynamics, fostering an environment where every team member feels empowered to contribute. A three-day multidisciplinary training in a Madagascar hospital led to 100% adherence. Participants reported increased awareness, improved communication and enhanced teamwork (68). Studies have demonstrated that surgical teamwork and compliance enhance results with highly effective teams attaining markedly lower rates of adverse events (69). A group of hospitals in Mogadishu, Somalia provided comprehensive training which included hands-on demonstrations, interactive sessions and the provision of instructional materials. After this intervention, adherence to the WHO SSC increased significantly. Compliance rates rose from 37% pre-intervention to 98.8% post-intervention, with the mean adherence score improving from 51.6% to 94.1%. This underscores the effectiveness of targeted training programs in enhancing surgical safety practices in resource-limited settings (43). The impact of interprofessional checklist briefings on communication breakdowns among surgical team members in the OR has indicated that briefings can help decrease the frequency of miscommunication, encourage proactive collaborative team communication and provide clinical motivation, all of which contribute to the reduction of errors (70, 71). The transformation of the local safety culture in the OR is essential to encourage team member communication, provide everyone the confidence to raise issues, and position everyone as a leader in patient safety (52).

**Figure 2:**
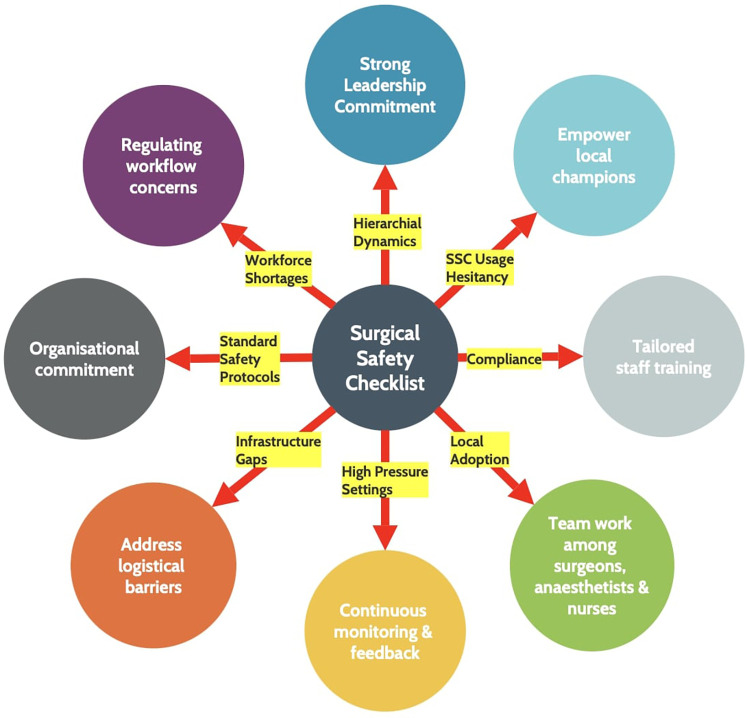
Barriers and implementation strategies for SSC usage.

Lastly, enthusiastic ‘local champions such as surgeons, anesthetists and nurses who are passionate about patient safety and willing to advocate for SSC usage should be included in the preliminary team. Eventually, those who are hesitant about the implementation of SSC will stop objecting and begin using the intervention in their practice after witnessing its successful use by local champions (52, 63). Training videos, developed and endorsed by senior surgical leaders, can provide clear guidance on checklist use and demonstrate effective teamwork. Workshops on checklist administration provided by trained and skilled surgical leaders can foster team spirit within local surgeons, anesthetists and nurses in addition to educating team members on their roles throughout the checklist protocol (63). Furthermore, the following five widely used domains—’train and educate stakeholders,’ ‘adapt and tailor to context,’ ‘provide interactive assistance,’ ‘develop stakeholder relationships,’ and ’support clinicians’—can help with the implementation of WHO SSC in LMICs (19). Process improvements are changes that make surgical care safer and more organized. These changes include standard safety and equipment checks, clear roles for team members, and better team communication but these improvements may not be as successful as they once were. The success of SSC depends on continued attention and quality control. Regular audits and feedback are therefore essential to catch problems early and keep the checklist effective, especially in high-pressure settings with staff shortages. Without audits, even proven tools like the SSC can lose value over time (Figure 2).

## Future directions

The SSC represents a low-cost, high-impact intervention that has been instrumental in strengthening surgical safety in LMICs. Despite its demonstrated value, SSC adoption remains inconsistent, partly due to the limited volume of implementation studies in LMICs compared to high-income countries (HICs). Future efforts should move beyond establishing the checklist's effectiveness in reducing complications and instead focus on strategies that ensure its sustained integration into routine surgical practice. This requires investment in structured implementation research, particularly mixed methods approach that address barriers such as entrenched hierarchies, time constraints, and resource limitations. Integrating digital platforms such as ’SurgHub’ into clinical workflows has also shown promise in enhancing adherence to safety protocols and improving patient outcomes (55). With the rise in Artificial Intelligence (AI) integration into healthcare systems, the Surgical Safety Checklist Module by Surgical Safety Technologies (SST) an AI-driven tool designed to objectively audit compliance with surgical safety protocols can be used wherever possible (72). Unlike traditional manual assessments, this module automates the capture of briefing, time-out and debriefing procedures, providing comprehensive analyses to indicate compliance rates and identify areas for improvement.

National-level initiatives where governments collaborate with surgical societies can further improve uptake. Examples of such initiatives include India's own National Patient Safety Implementation Framework (NPSIF) and the broader National Surgical, Obstetric, and Anesthesia Plans (NSOAPs) (73, 74). NSOAP has been developed for several LMICs such as Zambia, Tanzania, Nigeria, Ethiopia, Rwanda and Madagascar. India's NPSIF has highlighted surgical error reduction as a priority (75). The Global Patient Safety Action Plan 2021–2030 underscores the checklist's integral role and supports its implementation, monitoring, and evaluation to enhance patient safety globally (76). Additionally, the development of an updated and modified version of the SSC co-created with multidisciplinary stakeholders could incorporate components such as deep vein thrombosis (DVT) prophylaxis and hypothermia prevention, which are critical for minimizing preventable harm. South-to-South collaborations, learning and adaptation of best practices from other LMICs can serve to further strengthen implementation and long-term sustainability. Authors are currently conducting a qualitative analysis examining the views of surgeons and hospital staff on using the WHO SSC.

## Conclusion

The WHO SSC is a two-fold tool that is inexpensive and effective. Its consistent use reduces morbidity, mortality, complications and preventable harm by standardizing key practices. SSC's implementation has shown mixed results with roadblocks such as hierarchical dynamics, workforce shortages, limited training, language and cultural differences. However, strong leadership commitment, empowering local champions, tailored staff training, teamwork among surgeons, anesthetists and nurses, continuous monitoring and feedback, addressing logistical barriers, organizational commitment and ⁠regulating workflow concerns are vital parts of implementation and integration of SSC usage. The checklist offers a practical solution to strengthen and transform surgical systems in LMICs which can move from one-time adoption to sustained practice.
